# Influence of the Thermal Treatment to Address a Better Osseointegration of Ti6Al4V Dental Implants: Histological and Histomorphometrical Study in a Rabbit Model

**DOI:** 10.1155/2018/2349698

**Published:** 2018-06-27

**Authors:** Antonio Scarano, Ezio Crocetta, Alessandro Quaranta, Felice Lorusso

**Affiliations:** ^1^Department of Medical, Oral and Biotechnological Sciences and CeSi-MeT, University of Chieti-Pescara, Via dei Vestini 31, 66100 Chieti, Italy; ^2^Department of Medical, Oral and Biotechnological Sciences, University of Chieti-Pescara, Via dei Vestini 31, 66100 Chieti, Italy; ^3^Oral Health Centre of Western Australia, University of Western Australia, Perth, WA 6009, Australia

## Abstract

**Background:**

Pure titanium continues to be the first choice for dental implants and represents the gold standard for their biocompatibility and physical and mechanical characteristics, while the titanium alloy (Ti6Al4V) has good mechanical properties. The surface structure of the titanium oxide layer formation on the surface influences and improves the bone response around dental implants.

**Purpose:**

The purpose of this study is to evaluate the influence of a thermal treatment of Ti6Al4V implant surfaces and the bone healing response* in a rabbit model*.

**Methods:**

Altogether sixteen implants with same design were inserted into the distal femoral metaphysis. A screw (13 mm long, 4 mm in diameter) was inserted in an implant bed. Each rabbit received two implants, one in the left femur and one in the right femur. The samples were histologically** and histomorphometrically evaluated at 8 weeks**.

**Results:**

A statistically significant difference (*p* = 0.000034) was present histologically in the percentages of bone-implant contact (BIC) between the test group (BIC = 69.25±4.49%.) and control group (BIC = 56.25 ± 4.8%) by one-way analysis of variance (ANOVA). Significance was set at *p* ≤ 0.05.

**Conclusions:**

The outcome of the present study indicates a novel approach to improving bone healing around titanium implants.

## 1. Introduction

The clinical success of metallic biomaterials used in the substitution of teeth is based on a low toxicity, good long-term mechanical stability, and a high degree of osseointegration [1, 2]. In the past several decades, there was a strong interest in the surface properties of biomaterials [3]. Different approaches are being investigated to try to obtain an ideal implant surface that is conducive to bone formation in the peri-implant region [4]. The tissue response to biomaterials is influenced by nano-, micro-, and macrotopography of their surface [5]. It is well established that characteristics of implant surfaces, such as nano- and microtopography, and physicochemical composition positively influence the outcome of osseointegration, especially at the biological level, aiming at histological and morphological compatibilities [6].

Most probably there is optimal microroughness that affects the initial healing processes [7–9]. Different patterns of microtopography were used for quantitative description of implant surface topography, for example, arithmetical mean height of a line (Ra), and the difference in height of each point compared to the arithmetical mean of the surface (Sa).

Macro- and microtopography of an implant surface improve the contact surface between the implant and the bone and in turn the biomechanical connection between bone and implant.

The optimal surface roughness has not yet been determined, even though Han et al. have reported that a surface roughness of 1.5 *μ*m produced a stronger bone response than smoother or rougher surfaces [10]. A considerable variation exists in surface properties, such as topography, roughness, oxide thickness, oxide composition, and microstructure [11]. Current search to reduce the healing times of lifelong dental implants has focused on using surface-modification treatments [12, 13]. The efforts search for new surface treatments that are to get better bioactivity in order to facilitate the formation of hard tissue around it is increasingly important. Titanium's ability to endure the harsh bodily environment is a result of the protective oxide film that forms naturally in the presence of oxygen. The oxide film is chemically impermeable, insoluble, and strongly adherent and is preventing reactions between the titanium and the surrounding environment. A thin passivizing oxide scale is formed on titanium in ambient conditions, which inhibits release of Ti ions from the implant surface.

The native oxide that forms on titanium surfaces upon exposure to air is TiO_2_, but lower oxidation states such as Ti_2_O_3_ and TiO have also been observed to exist in ambient conditions [14, 15]. Titanium is naturally passivated, forming an oxide film that becomes polarized and heterogeneous as a result of exposure over time to bodily environments. Titanium with stable oxide layers, especially consisting of TiO_2_, gets better wetting of the implant in contact with biological fluid [16]. To achieve faster osseointegration, the surface of titanium implant samples was subjected to thermal treatment. The aim of this study is to examine the influence of thermal treatment of Ti6Al4V dental implants on bone healing in rabbits.

## 2. Materials and Methods

Threaded Ti6Al4V dental implants (4 mm diameter and 10 mm length) were used in the present study (ISOMED Implant System, Italy). The textured Ti6Al4V dental implants surfaces were obtained through acid-etching without grit-blasting of plateau root form endosseous Ti6Al4V bulk alloy implants of 4 mm in diameter by 10 mm in length The oxidation treatment was performed at 802°C for 1 min in the air. The test implants were placed at the center of the furnace and connected to a temperature controller to maintain a working temperature of 802°C. 802°C was chosen because at this temperature there is increase in crystalline anatase, while short time was chosen to reduce mechanical weak risks [17]. After thermal treatment, Ti6Al4V dental implants were removed from the furnace and cooled in distilled water. The oxidation treatment was performed on the test implants immediately before implant placement.

### 2.1. Implant Placement

The protocol was approved by the local Ethical Committee of University of Chieti-Pescara, Italy. Eight skeletally pathogen-free (SPF) and virus antibody-free (VAF) mature male New Zealand white rabbits (Crl:KBL(NZW), 9 months old and 3.5 Kg weight) obtained from Charles River Laboratories (Lieu-dit Oncins, France) were used in this study.

Altogether sixteen implants with same design were inserted into the distal femoral metaphysis. A screw (13 mm long, 4 mm in diameter) was inserted in an implant bed. Each rabbit received two implants, one in the left femur and one in the right femur.

The animals were anesthetized with intramuscular injections of fluanisone (0.7 mg/kg b.wt.) and diazepam (1.5 mg/kg b.wt.) and local anesthesia; 1 ml of 2% lidocaine/adrenalin solution was applied. A skin incision with a periosteal flap was used to expose the bone surface. The osteotomies were prepared with a 2 mm pilot bur used on an implant motor machine (Esacrom, Imola, Italy) operated at 600 rpm with saline irrigation.

The periosteum and fascia were sutured with polyglycolic acid and the skins with silk (Sweden & Martina, Italy). Analgesics with tramadol hydrochloride (Altadol, Abiogen Pharma S.P.A, Italy) and oxytetracycline dihydrate (Terramicina Long Acting by Pfizer Italia srl), 100 mg/kg, single dose, were given for 1 week. After 2 weeks after surgery, the sutures were removed. Postsurgical visits were scheduled daily to check the course of healing. No rabbit deaths or complications occurred in the postoperative period. All rabbits were euthanized with an overdose of intravenous pentobarbital at 8 weeks. A total of 16 implants were retrieved.

### 2.2. Specimen Processing

Implants and surrounding tissues were washed in physiological solution and immediately fixed in 4% paraformaldehyde and 0.15 M cacodylate buffer in 0.1% glutaraldehyde at 4°C and pH 7.4, to be processed for histomorphometry. The implants were processed to obtain thin ground sections with the Precise 1 Automated System (Assing, Rome, Italy). They were previously dehydrated in an ascending series of alcohol rinses and embedded in a glycol methacrylate resin (Technovit 7200 VLC, Kulzer, Wetzlar, Germany). The specimens after polymerization were sectioned, along their longitudinal axis, with a high-precision and accuracy diamond disc at about 150 *μ*m and ground down to about 30 *μ*m with a specially designed grinding machine. A total of 2 slides were obtained for each specimen. The slides were stained with toluidine blue and acid fuchsin. They were observed in normal transmitted light under a Nikon microscope ECLIPSE (Nikon, Tokyo, Japan). The percentage of hard tissues in close contact with implant was determined using a light microscope connected to a high-resolution video camera (16.25-megapixel) (Digital Sight series microscope cameras) and interfaced to a monitor and computer (Notebook Toshiba Satellite Pro r50-c-15w). This optical system was associated with a digitizing pad (Matrix Vision GmbH) and a histometry software package with image capturing capabilities (Image-Pro Plus 4.5, Media Cybernetics Inc., Immagini & Computer Snc, Milano, Italy).

Two implants for each group were analyzed under a Leo scanning electron microscope (Zeiss, Hallbergmoos, Germany).

#### 2.2.1. Statistical Evaluation

A power analysis was made using clinical software, freely available on the site http://clincalc.com/stats/samplesize.aspx, for determining the number of implants needed to achieve statistical significance for quantitative analyses of histomorphometry. A calculation model was adopted for dichotomous variables (yes/no effect) by putting the effect incidence designed to caution the reasons, 10% for controls and 95% for treated group. The significance level (alpha) was set at 0.05 and power at 80%. The optimal number of implants for analysis is 8 implants.

Differences between the groups of treatment were analyzed by one-way analysis of variance (ANOVA) followed by Fisher's Protected Least Significant Difference (PLSD) post hoc test. A *p* value < 0.05 was considered statistically significant. Data treatment and statistical analysis were done by Excel origin and SPSS software.

## 3. Results

### 3.1. Light Microscopy

#### 3.1.1. Control Implant

Microscopically, all 8 implants were well integrated into bone. No fibrous tissue was observed between bone and implant surfaces in all implants.

**Figure 1:**
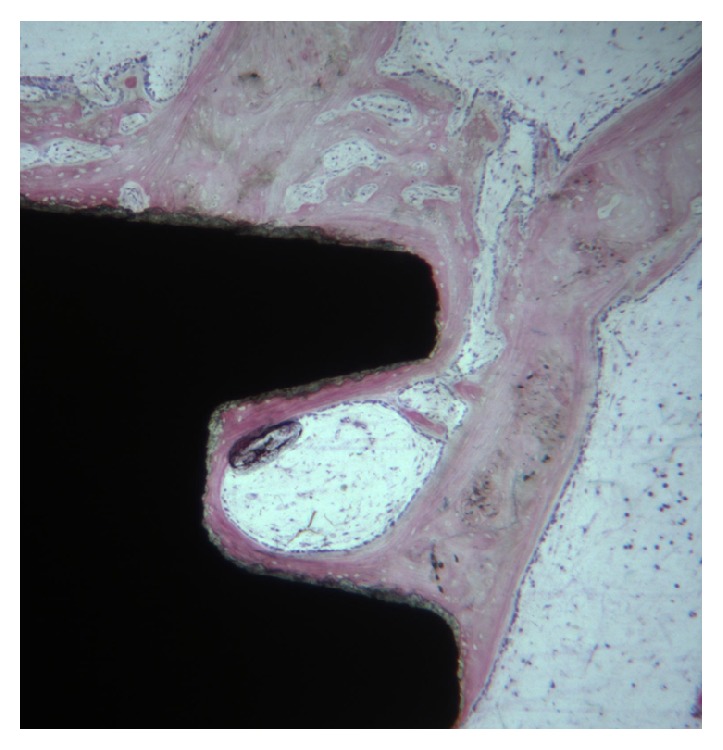
The bone formation was observed in the cortical portion of implant. Toluidine blue and acid fuchsin 20X.

Newly formed bone was found in close contact with the implant surface (Figure 1). The bone trabeculae were wide and contained large osteocyte lacunae. The osteoblasts were actively secreting the osteoid matrix that, in some areas, was undergoing mineralization.

**Figure 2:**
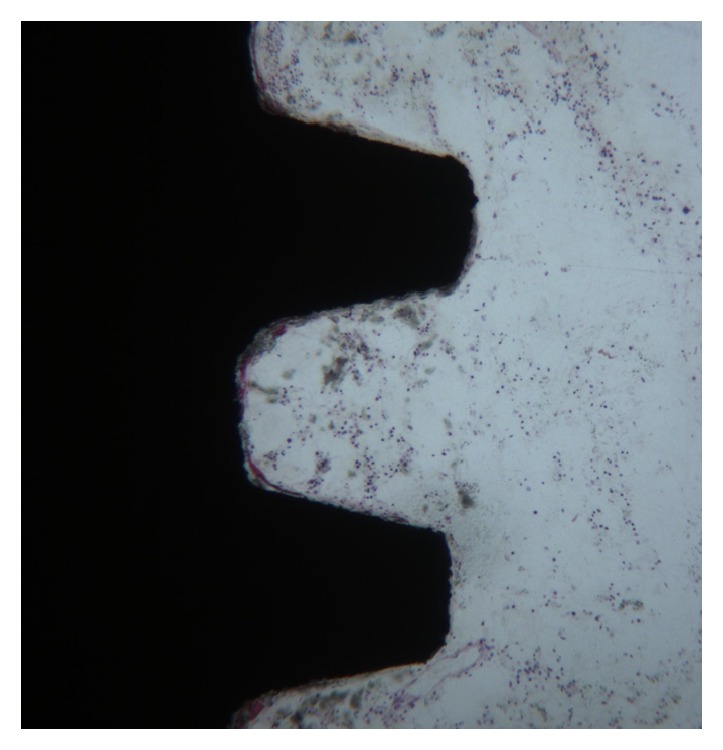
Newly trabecular bone is present in close contact with implant surface. Toluidine blue and acid fuchsin 50X.

**Figure 3:**
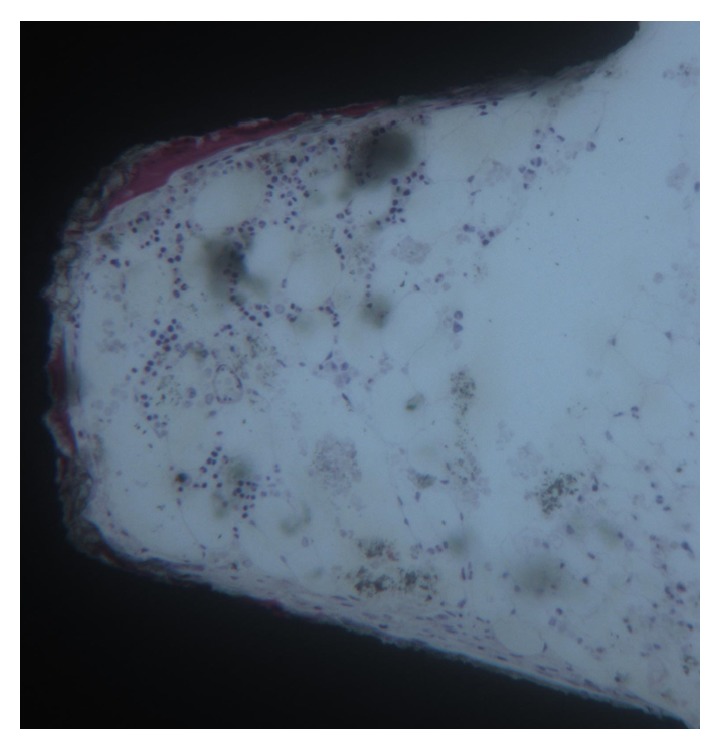
At higher magnification, it is possible to observe newly trabecular bone in the medullary space. Toluidine blue and acid fuchsin 100X.

Only a few osteoblasts were present (Figures 2 and 3). Bone formation was observed in close contact with implant surface. Mature mineralized bone and, only in a few areas, not yet mineralized osteoid matrix were detected at the interface. The bone-implant contact percentage was 56.25 ± 4.8%.

#### 3.1.2. Test Implant

In general, bone morphology presented differentiated cellular lines specific of mineralized bone, such as osteoblasts, osteocytes but also osteoid, and blood vessels.

**Figure 4:**
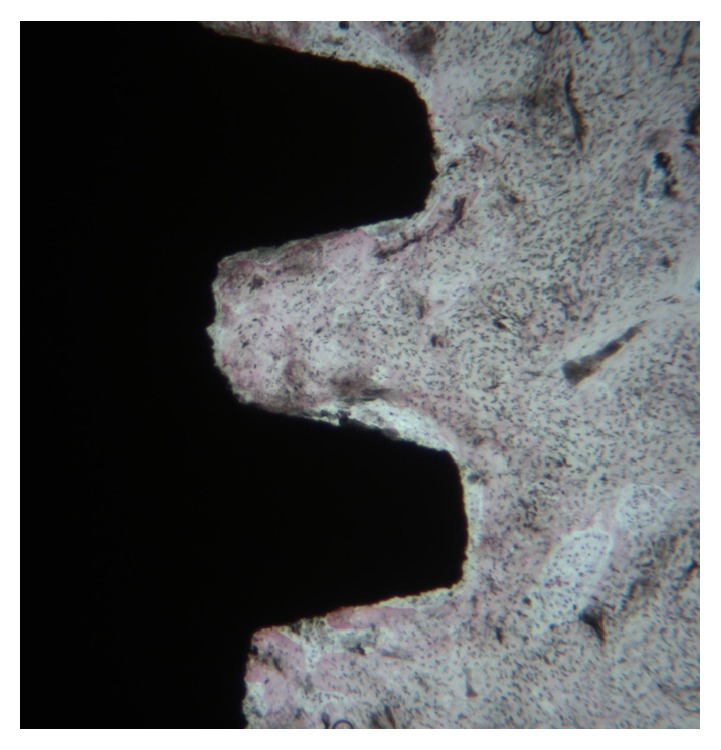
Mature bone and immature primary osteons (PO) were visible in coronal portion of implant. Toluidine blue and acid fuchsin 20X.

**Figure 5:**
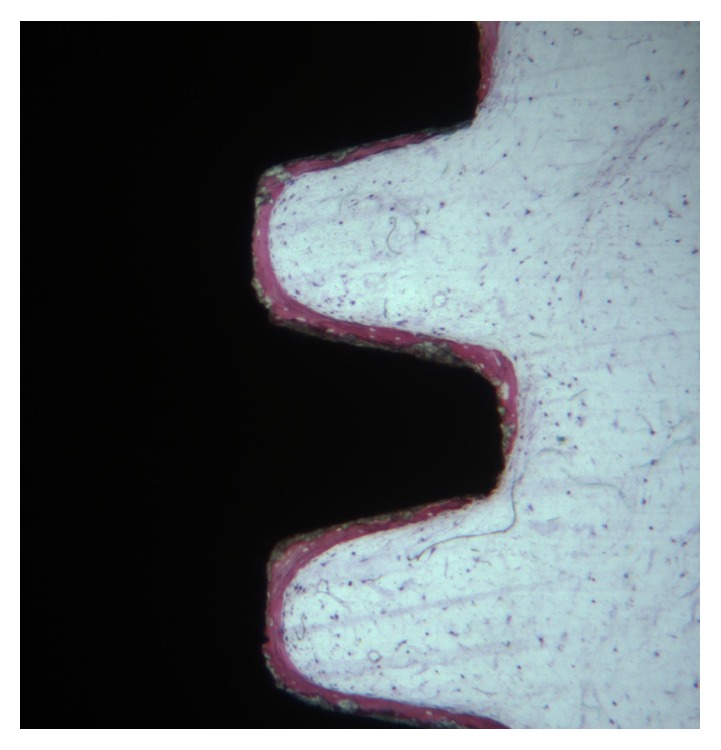
Newly trabecular bone in the contact to all implant surface. Toluidine blue and acid fuchsin 50X.

**Figure 6:**
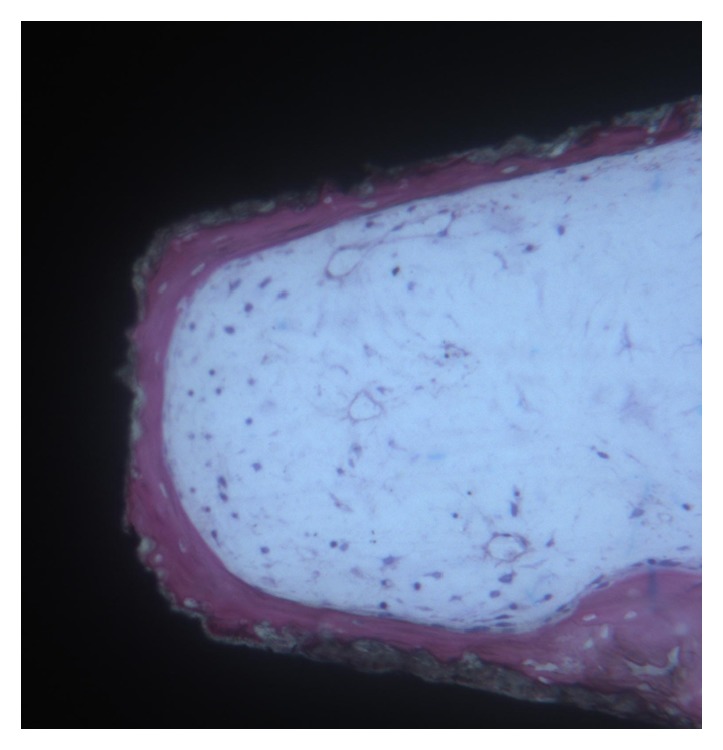
At higher magnification, it is possible to observe trabecular bone in direct contact of the thread concavities. Toluidine blue and acid fuchsin 100X.

Microscopically, all 8 implants were well integrated into bone (Figures 4–6). Fixtures were in contact with cortical bone along the upper threads, while the lower threads were in contact either with newly formed bone or with marrow spaces. Soft tissue was absent between bone and titanium surfaces in all implants.

It was possible to observe a large number of newly formed bone trabeculae that were in contact with the implant surface (Figures 4–6). A few osteoblasts were actively secreting the osteoid matrix that, in some areas, was undergoing mineralization. The absence of necrotic processes of the preexisting bone when in contact with the implants was clearly observed. The bone-implant contact percentage was 69.25±4.49%.

#### 3.1.3. Statistical Analysis

The histological results showed bone-implant contact percentages on both implant surfaces. A statistically significant difference was found in the percentages of bone that had formed in the test group compared to the control group implants (*p* = 0.000034).

## 4. Discussion

The outcomes of the present research showed that the implant surface modified by thermal treatment increases the bone implant contact.

In fact, a higher difference was found in the percentages of bone implant contact that had formed on the implants modified by thermal treatment than on the control implants after eight weeks.

Different surface roughness [18, 19], as well as oxidation to form thicker or otherwise modified TiO2 layers on the surface [20], has been found to be beneficial for the biological performance of the implants. In this in vivo study, we have shown that the use of implant modified by thermal treatment accelerates the osseointegration process.

When exposed to air or liquids, titanium produces a layer of oxide which reduces its reactivity, and this oxide layer interacts with the tissues [21]. Titanium dioxide is a very versatile and robust material that has been used in many industrial applications, such as photocatalysis and solar energy conversion [22], pigment in food paints, cosmetics, ceramics, sunscreen, and toothpaste [7]. Titanium dioxide exists in three different crystal lattices, anatase, rutile, and brookite [23]. Rutile is the thermodynamically favoured phase and it has been shown to be more stable than anatase at low and high pH levels [24].

The implant surface can be described as “bioactive” when it promotes the formation of hydroxyapatite on the surface in contact with body fluids [25]. There are several materials that are known to be bioactive, but almost all of them are resorbable* in vivo* [26]. Recent studies have also convincingly demonstrated that calcium- and phosphate-based coatings on Ti surfaces further increase the osseoconductivity obtained through texturing Ti surfaces alone [27]. In addition to the effects of surface topography and surface chemistry, thin depositions of hydroxyapatite (HA) and calcium phosphate (CaP) crystals on implant surfaces have been shown to accelerate early bone formation and increase the strength of the bond between implant and bone [28]. TiO_2_ has bioactive properties and promotes the formation of hydroxyapatite on the surface in contact with body fluids [29] and can be increased in thickness by heat treatment [30], sol-gel coatings [31], and the physical-vapour-deposition (PVD) of titanium oxide [32]. Also autoclaving of implant increases the apatite deposition at interface [33] and this latter phenomenon has been long correlated to osteointegration ability of implants [34]. Moreover, UV irradiation on titanium implant enhances bone integration and increases the TiO_2_ layer thickness [35]. Several techniques, including anodic oxidation [36–39] and thermal treatment [40–43], were used to achieve the deposition of the titanium oxide for improving osseointegration or prevent bacterial adhesion. The TiO_2_ layer on the transmucosal portion of the abutment reduces the quantity of bacteria that attach to the metal surface and produces more healthy peri-implant tissues [43]. Moreover, the TiO_2_ coating could be hypothesized, with positive effects in cases of peri-implant crestal bone resorption during peri-implantitis, when a coating that decreases the bacterial charge could be helpful in the treatment of peri-implant disease.

During peri-implant infection, the microorganisms through involved cytokines (interleukins 1*β*, 6, and 10 and TNF-*α*) could contribute to bone loss in peri-implantitis [44].

The rabbit was chosen as an animal model because it is a convenient model for skeletal research studies [45] and has been extensively used to test the osteoconductive/osteoinductive reaction to implant biomaterials [46]. In addition, the rabbit model provides an excellent cost-effective animal model; its maintenance and housing are simple and it recovers very well postoperatively. Similarity of bone composition between rabbit and human bone exists [47].

In this study, we chose a femur model to determine the potential ability of implant surfaces to enhance bone formation when modified by thermal treatment in the presence of large medullary spaces. In this site, the new bone formation was easily evaluated without the interference of native bone. In fact, the coronal portion of the implant was located within the cortex, while the remaining part of the implant protruded into the marrow cavity, without contacting the endosteal surface of the opposite cortex. In the present study, more bone implant contact (BIC) was found in the treated group than in the control group.

All the implants used in this research were similar with regard to length and diameter and chemical surface composition but differed in terms of surface oxide thickness, surface topography, and crystal structure. The thermal treatment at 700°C for 1 h of the dental implant does not change the roughness surface at microscale of specimens [48]. Therefore, the increase in BIC is due to the properties of the TiO_2_ layer. One plausible explanation is that the thermal treatment had influenced very early events such as biomolecule adsorption, which in turn could influence cellular reactions and the ensuing development of tissue with early deposition of newly formed bone, which is in direct contact with the implant surface.* In vitro* experiments with primary osteoblast cell culture revealed that the surface modification does not alter but may improve the excellent biocompatible behaviour. In fact, cell adhesion and osteointegration are favored on the thermally treated surfaces [49, 50].

In conclusion, these results show that surfaces modified by thermal treatment improve bioactivity and BIC compared to those control implants and could be clinically advantageous for shortening the implant healing period for implants placed in areas with low-density bone.

## Data Availability

All data used (bone-implant contact percentages) to support the findings of this study are available from the corresponding author upon request. The authors have annotated the entire data building process and empirical techniques presented in the paper.
