# Corrigendum to “Influence of the Thermal Treatment to Address a Better Osseointegration of Ti6Al4V Dental Implants: Histological and Histomorphometrical Study in a Rabbit Model”

**DOI:** 10.1155/2018/3408676

**Published:** 2018-11-01

**Authors:** Antonio Scarano, Ezio Crocetta, Alessandro Quaranta, Felice Lorusso

**Affiliations:** ^1^Department of Medical, Oral and Biotechnological Sciences and CeSi-MeT, University of Chieti-Pescara, Via dei Vestini 31, 66100 Chieti, Italy; ^2^Department of Medical, Oral and Biotechnological Sciences, University of Chieti-Pescara, Via dei Vestini 31, 66100 Chieti, Italy; ^3^Oral Health Centre of Western Australia, University of Western Australia, Perth, WA 6009, Australia

In the article titled “Influence of the Thermal Treatment to Address a Better Osseointegration of Ti6Al4V Dental Implants: Histological and Histomorphometrical Study in a Rabbit Model” [1], the method description was incomplete and inaccurate. The manufacturer name for the threaded Ti6Al4V dental fixtures that were used in the present study was Bicon Implants (Bicon, Boston, MA United States). The outcome, the histologies and the scientific evidence that emerged from the study must be considered in relation to the properties of the medical device tested in the experiment. In addition, the length of the implant size of the Threaded Ti6Al4V was incorrectly given as “10 mm”, while it should be corrected to be “11 mm”.

Therefore, “Threaded Ti6Al4V dental implants (4 mm diameter and 10 mm length) were used in the present study (ISOMED Implant System, Italy). The textured Ti6Al4V dental implants surfaces were obtained through acid-etching without grit-blasting of plateau root form endosseous Ti6Al4V bulk alloy implants of 4 mm in diameter by 10 mm in length” should be corrected to “Threaded Ti6Al4V dental implants (4 mm diameter and 11 mm length) were used in the present study (Bicon Implants, Bicon, Boston, MA United States). The textured Ti6Al4V dental implants surfaces were obtained through acid-etching without grit-blasting of plateau root form endosseous Ti6Al4V bulk alloy implants of 4 mm in diameter by 11 mm in length”.
